# Surface-Engineered Magnesium Metal–Organic Frameworks for Curcumin Stabilization: Interfacial Coordination Enhances Aqueous Stability and Boosts Antioxidant Efficacy 32-Fold

**DOI:** 10.1021/acsomega.6c06121

**Published:** 2026-08-17

**Authors:** Pamela Al Azzi, Riham El Kurdi, Joelle Mesmar, Mohamad Hmadeh, Digambara Patra

**Affiliations:** † Department of Chemistry, 11238 American University of Beirut, P.O. Box: 11-0236, Riad El Solh, Beirut 1107−2020, Lebanon; ‡ Department of Biology, American University of Beirut, P.O. Box: 11-0236, Riad El Solh, Beirut 1107−2020, Lebanon

## Abstract

Curcumin possesses exceptional therapeutic and antioxidant potential, yet its clinical translation remains severely bottlenecked by rapid hydrolytic degradation and poor stability in aqueous environments. Herein, we report a robust and highly efficient magnesium-based metal–organic framework (MgMOF) platform designed to encapsulate and stabilize curcumin via a facile wet impregnation strategy. Systematic optimization of processing parameters—including kinetics, thermodynamics, and mass ratios—revealed that a 1 h stirring regime with a 1:4 MgMOF-to-curcumin molar ratio yields a superior loading capacity of 46 wt % while preserving the long-range crystalline order of the host framework. Comprehensive structural and physiochemical characterization utilizing thermogravimetric analysis, powder X-ray diffraction, scanning electron microscopy, and Fourier-transform infrared spectroscopy confirmed successful surface adsorption driven by specific coordination interactions between the curcumin functional groups and the open Mg^2+^ coordination centers. Crucially, the MgMOF-curcumin conjugate demonstrated a 4-fold reduction in the curcumin degradation rate, maintaining structural integrity over an extended 10-day period in aqueous media. Furthermore, 2,2-diphenyl-1-picrylhydrazyl radical scavenging assays revealed that the host matrix not only preserves but also synergistically boosts bioactivity, culminating in an unprecedented 32-fold enhancement in antioxidant efficacy at physiologically relevant neutral pH compared to free curcumin. These findings highlight the potential of MgMOFs as biomaterial carriers to overcome the intrinsic limitations of hydrophobic bioactives, paving the way for advanced pharmaceutical and targeted therapeutic delivery systems.

## Graphical abstract

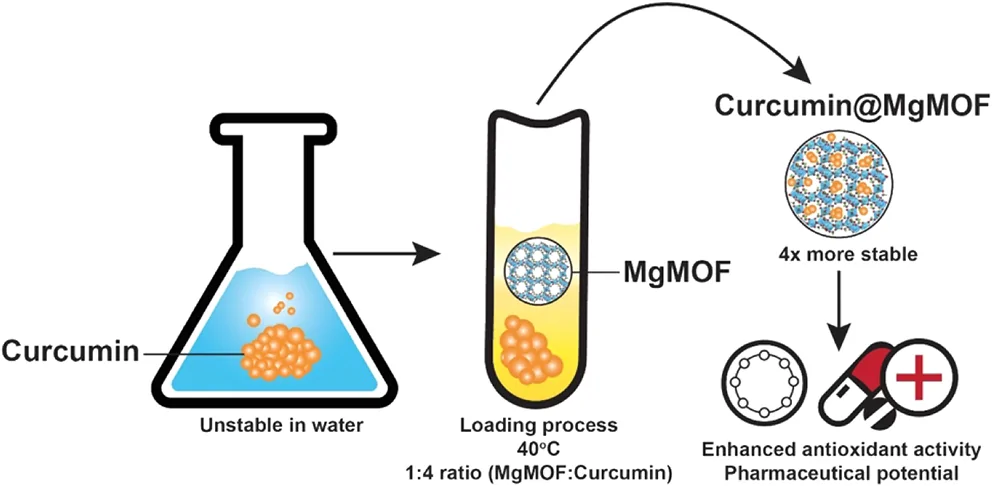

## 1 Introduction

Antioxidants are compounds capable of delaying or inhibiting oxidation by donating electrons or hydrogen atoms to free radicals, thereby neutralizing these reactive species and preventing oxidative damage. Their radical-scavenging activity is strongly influenced by their molecular structure, particularly the number and position of hydroxyl groups on aromatic rings.^1^ Owing to their ability to inhibit oxidative degradation, antioxidants are widely employed across diverse fields to improve product stability and prolong shelf life. Synthetic antioxidants such as *tert*-butylhydroquinone (TBHQ), butylated hydroxyanisole (BHA), and butylated hydroxytoluene (BHT), are commonly used as food preservatives^2^ and as stabilizing agents in pharmaceutical formulations.^3^ However, the chronic use of synthetic antioxidants has raised increasing health concerns due to reports of potential adverse effects, including DNA damage,^4^ gastrointestinal tract problems, liver damage, and increased risk of cancer.^5^ In addition, the environmental fate of these synthetic agents remains insufficiently investigated.^6^ These concerns have stimulated growing interest in the study of naturally occurring antioxidants,^7^ with curcumin emerging as one of the most promising candidates.^8^

Curcumin, a crystalline yellow natural polyphenol is the main component of turmeric which is derived from the dried rhizomes of *Curcuma longa*.^9^ With a molecular formula of C_21_H_20_O_6_, and the chemical name diferuloylmethane,^10^ curcumin is known to be the “Wonder drug of life”.^11^ Besides its traditional and global use as a flavoring and coloring agent, curcumin is recognized for its therapeutic properties including anticancer,^12^ antioxidant,^13^ and anti-inflammatory activities.^14^ However, despite its remarkable biological potential, the clinical application of curcumin is still limited due to its low bioavailability acquired by its poor solubility and chemical instability.^15^

To enhance its water solubility, improve its stability and overcome its poor bioavailability, curcumin has been loaded into various hosts, including liposomes,^16^ polymers,^17^ and metal–organic frameworks (MOFs).^18^ MOFs are a crystalline class of materials constructed by metal clusters linked with organic ligands, forming well-defined three-dimensional porous networks.^19^ Their high surface area, permanent porosity, and tunability,^20^ make MOFs useful in various applications such as catalysis,^21^ drug delivery,^22^ and wastewater treatment.^23^ Among the reported MOF materials, Mg_3_(HCOO)_6_ (also known as magnesium formate) is a well-known microporous MOF that is commercially available under the trade name Basosiv M050. It can be easily synthesized on a large scale through a solvent-free approach, employing inexpensive and nontoxic precursors, such as magnesium oxide and formic acid. This MOF exhibits a high stability in a range of organic solvents, and maintains structural integrity even after activation at temperatures as high as 400 °C. The structure consists of one-dimensional channels with pore dimensions of 4.5 × 5.5 Å, decorated with oxygen atoms and C–H bonds (Figure 1).^24^ These structural features provide a suitable environment for curcumin loading.

**Figure 1:**
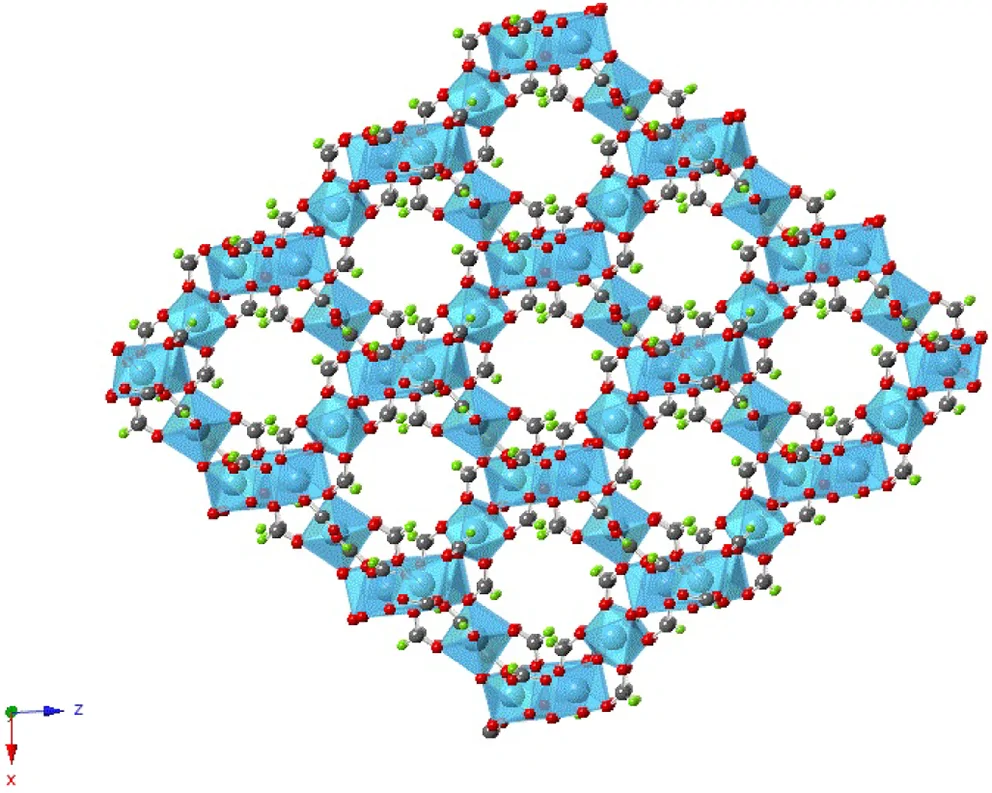
Crystal structure of magnesium formate metal organic framework (blue: magnesium, red: oxygen, gray: carbon).

Various methods have been developed to evaluate the antioxidant capacity of compounds. Among them, the 2,2′-diphenyl-1-picrylhydrazyl (DPPH) radical scavenging assay is one of the most widely employed. This method measures the ability of antioxidant molecules to donate electrons or hydrogen atoms to the stable nitrogen-centered DPPH radical.^25^ This reaction will result in the reduction of the purple DPPH radical to a yellow stable diamagnetic molecule,^26^ which is reflected by a decrease in absorbance at the characteristic wavelength of the DPPH radical. This decrease is proportional to the radical scavenging capacity of the candidate molecule.^27^

Earlier, we reported aluminum fumarate MOFs for improving curcumin’s stability and antioxidant activity.^28^ Herein, MgMOF is used as a matrix for curcumin loading. The wet impregnation technique is optimized to immobilize curcumin onto the surface of this framework. The resulting composite is characterized using various spectroscopic and microscopic techniques. Curcumin stability is then evaluated over 10 days to assess the effect of the loading on limiting its degradation. Finally, the scavenging activity of curcumin in the designed system is investigated using the DPPH radical scavenging assay.

## 2 Materials and Methods

### 2.1 Materials

Curcumin (C_21_H_20_O_6_), Magnesium Formate (C_2_H_2_MgO_4_), and 2,2-diphenyl-1-picrylhydrazyl (DPPH) were acquired from Sigma-Aldrich. Methanol (CH_3_OH) was purchased from Honeywell Riedel-de Haën. Buffer solution pH 4 (phthalate) and Buffer solution pH 7 (phosphate) were purchased from Fisher Chemical. All chemicals were used as obtained without further purification.

### 2.2 Curcumin Loading into MgMOF

Curcumin was immobilized onto the surface of MgMOF following the wet impregnation technique in which 25 mg of the MOF (0.22 mmol) was added to 55 mg (0.15 mmol) of curcumin in methanol (Scheme 1). The vial was then covered with aluminum foil and the mixture was stirred at 40 °C for 1 h. The product was recovered by removing the solvent under reduced pressure using a rotary evaporator, and was then washed twice with methanol, until a clear supernatant was obtained. Finally, the product was dried at 60 °C in a vacuum oven.

**Scheme 1:**
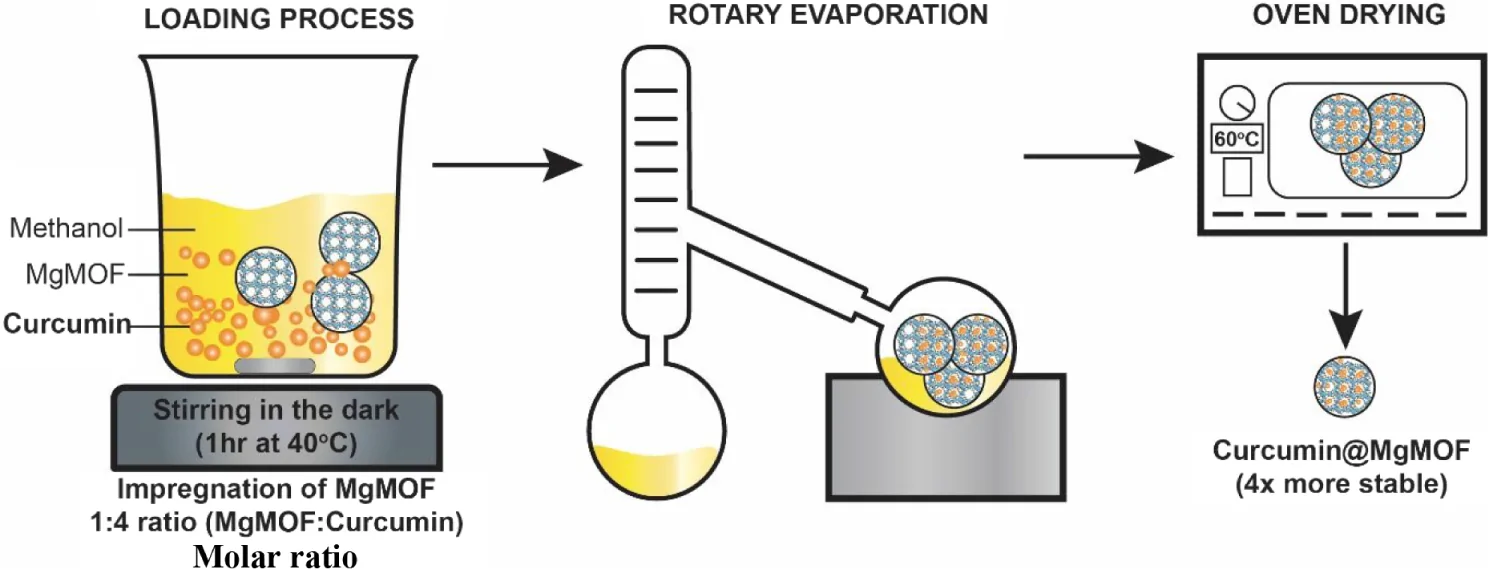
A Schematic Illustration of the MgMOF-Curcumin Preparation

### 2.3 Optimization of the Encapsulation Procedure

The reaction parameters described in Section 2.2 were optimized to obtain the most stable MOF-Cur system and maximize the loading efficiency. Accordingly, the procedure was repeated while varying one parameter at a time, recovering the product, and characterizing it. The investigated parameters included the time of stirring, temperature, and the MOF:Curcumin ratio.

### 2.4 Time Stability and Curcumin Degradation

To evaluate the effect of immobilization on the stability of curcumin, the degradation percentage of the immobilized curcumin was monitored over a period of 10 days by measuring its UV–vis absorbance at regular intervals. The measurements were performed in triplicate.

### 2.5 Characterization

To acquire information about the crystallography of our samples, they were placed on the zero-background holder and powder X-ray diffraction patterns were recorded using D8 advance X-ray diffractometer by Bruker. In addition, Thermogravimetric analysis was performed using (TGA 209) to evaluate the thermal decomposition of the samples under N_2_ atmosphere in a temperature range from 30 to 900 °C with 15 K/min step size. Data was evaluated using Netzsch software. Scanning Electron Microscopy (SEM) imaging was used to examine the size and shape of solid samples which were deposited on an aluminum holder coated with carbon conductive tape. It was performed by a MIRA3 LMU with OXFORD EDX detector, by Tescan. Finally, Bruker Tenor 27 FTIR was employed to obtain the Fourier-Transform Infrared Attenuated Total Reflectance (FTIR-ATR) spectra.

### 2.6 Antioxidant Activity

The DPPH radical scavenging assay was employed to evaluate the antioxidant activity of the MgMOF-Cur systems. For this purpose, samples having a fixed DPPH concentration (0.04 mg/mL) were prepared and increasing concentrations of MgMOF-Cur composite (0.01 mg/mL; 0.02 mg/mL; 0.03 and 0.04 mg/mL) were added. The mixtures were incubated for 1 h to allow the reaction to occur, after which the absorbance was quantified at 540 nm. The same steps were performed using free curcumin instead of the MOF-Cur system.

In order to test the effect of the pH of the medium on the antioxidant activity of curcumin, the same procedure for the DPPH radical scavenging assay was repeated. However, the MgMOF-Cur solutions were prepared in buffers of pH = 4 and pH = 11. All experiments were performed in triplicate.

## 3 Results and Discussion

### 3.1 Optimization of Curcumin Loading into MgMOF

Curcumin was loaded on the surface of MgMOF according to the simple wet impregnation technique^29^ which consists of adding MgMOF to a curcumin solution in methanol and stirring the mixture under controlled temperature and time conditions. The parameters of this procedure were subsequently optimized.

#### 3.1.1 a) Optimization of the Stirring Time

To be able to specify the optimal stirring time, three samples were prepared, and stirred respectively for 1 h (MgMOF-Cur 1), 24 h (MgMOF-Cur 2), and 48 h (MgMOF-Cur 3). The recovered composites were then characterized by thermogravimetric analysis (TGA) and powder X-ray diffraction (PXRD).

As shown in Figure 2A, pure curcumin underwent a major mass loss from 200 to 400 °C.^30,31^ According to Nagabhushana et al. MgMOF possesses a two-step mass loss.^32^ Similar data were obtained, as depicted in (Figure 2A), where the first mass loss occurred between 120 and 200 °C (∼20%), and is attributed to the loss of the water composition,^33^ while the second mass loss was observed around 400 °C (∼45%) and is assigned to the decomposition of curcumin.^34^ Upon curcumin loading onto the MOF, its mass loss was significantly reduced for the sample stirred for 1 h, highlighting the role of the MOF in enhancing the thermal stability of curcumin. This observation is consistent with previous studies reporting improved thermal stability of curcumin upon encapsulation within MOF systems.^35^ While the MgMOF-Cur 1 maintained similar thermal stability compared to the unloaded MgMOF, MgMOF-Cur 2 and MgMOF-Cur 3 which were obtained by longer stirring times, showed higher mass losses, suggesting that extended affected the structural integrity of the MOF.

**Figure 2:**
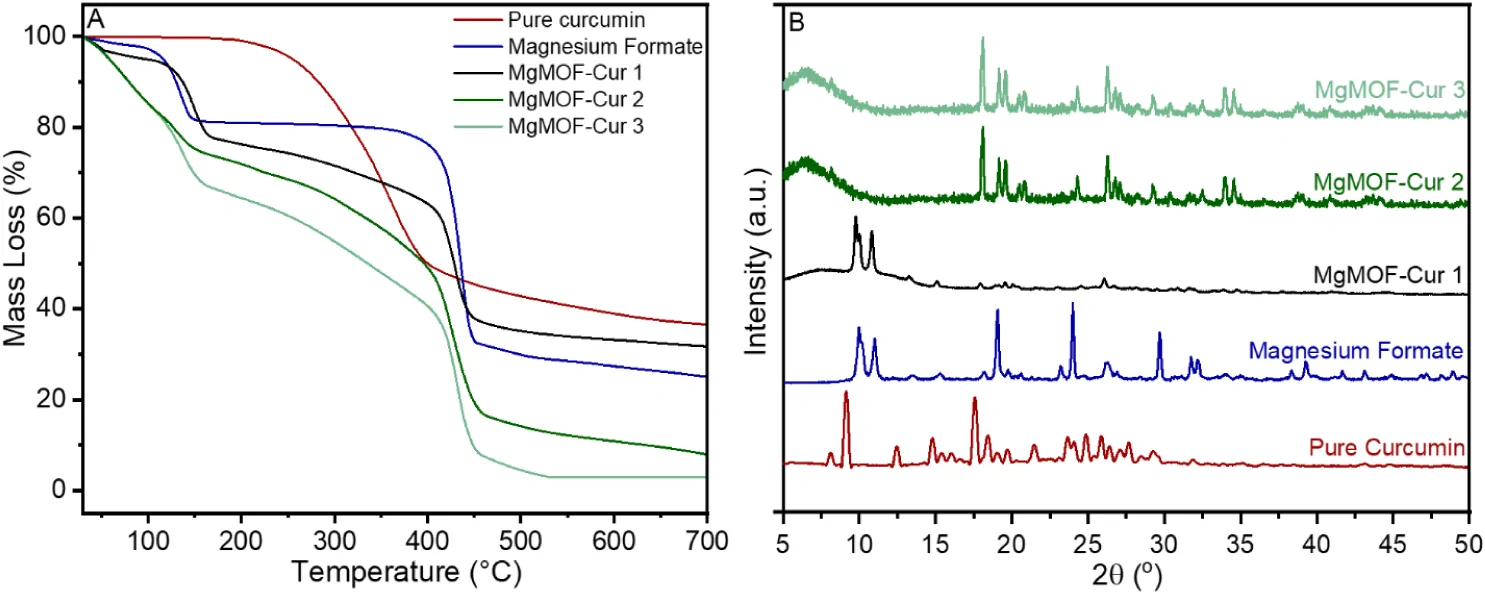
(A) TGA profiles and (B) PXRD diffractograms of pure curcumin, magnesium formate, and curcumin loaded onto magnesium formate with different stirring times (1 h, MgMOF-Cur 1; 24 h, MgMOF-Cur 2; and 48 h, MgMOF-Cur 3).

In addition, the narrow one-dimensional zigzag channel of MgMOF (∼4.9 Å),^24^ which are considerably smaller than the dimensions of a curcumin molecule, suggest that curcumin is preferentially adsorbed on the surface rather than incorporated into the pores. This interpretation is supported by the PXRD patterns shown in Figure 2B. The diffraction peaks from 10° to 30° 2θ correspond to crystalline curcumin.^36^ Remarkably, after curcumin loading onto MgMOF, the main peaks of the MOF were still observable while other peaks of the framework were hindered, suggesting the presence of curcumin on the surface shielding the diffraction from specific planes. Moreover, it was evident that the characteristic peaks of MgMOF, which are normally present at 9.8 and 10.8°,^37^ were only maintained in the MgMOF-Cur sample stirred for 1 h, while they disappeared in the diffractograms of the samples stirred for 24 and 48 h. This loss of crystallinity is in line with the TGA results and supports our previous suggestion that the MOF was destroyed upon prolonged stirring. Therefore, 1 h of stirring was selected as the optimal time for curcumin loading.

#### 3.1.2 b) Temperature Optimization

To assess the effect of heating on composite formation, two MgMOF-Cur samples were prepared by stirring the mixture at room temperature and at 40 °C, respectively. The results demonstrated improved thermal stability of curcumin in the system upon heating, as indicated by a lower mass loss observed for the heated mixture (Figure 3A). This improvement is likely associated with the increased curcumin loading resulting from enhanced solubility at elevated temperature.

**Figure 3:**
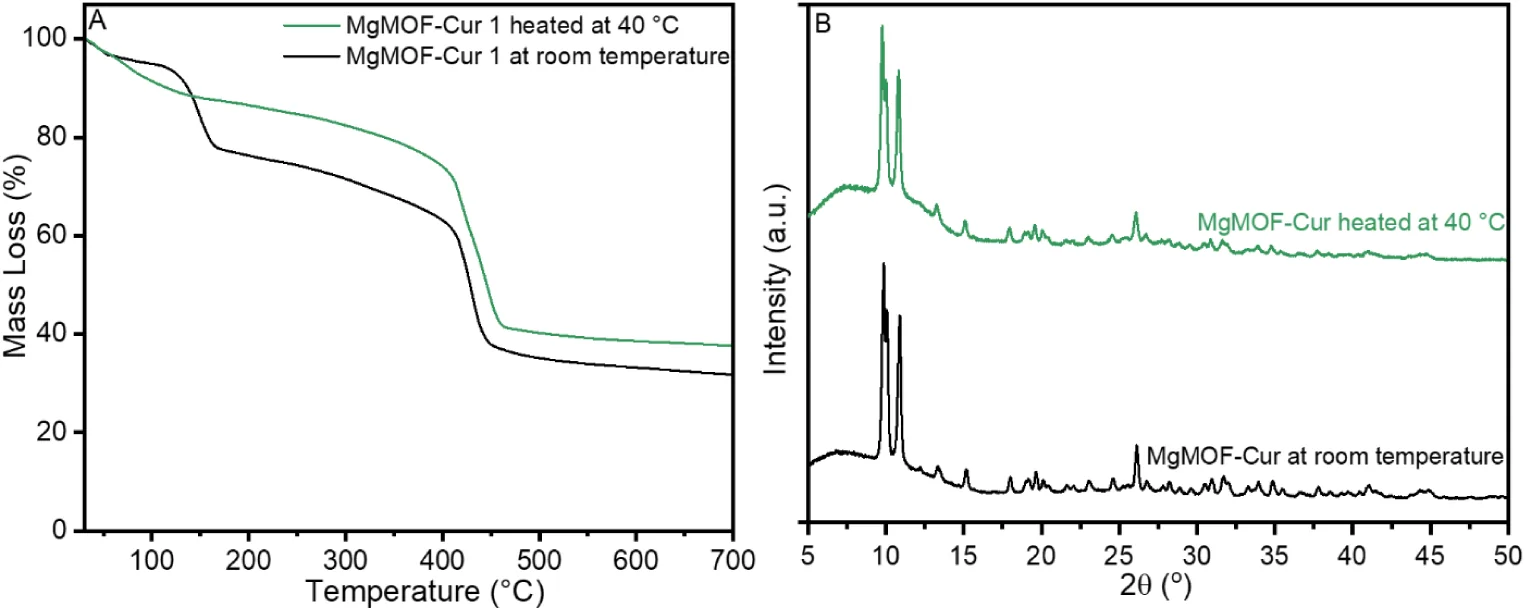
(A) TGA profiles and (B) PXRD diffractograms of curcumin loaded onto magnesium formate at room temperature and at 40 °C. (C) Optimization of the MgMOF:Curcumin ratio.

Based on the PXRD data (Figure 3B), the heating did not have any effect on the characteristic diffraction peaks. However, the crystallinity of the samples was affected (Table 1).

**Table 1:** Percentage Crystallinity of Pure Curcumin, MgMOF, and MgMOF-Cur in the Absence and Presence of Heat

	Curcumin	MgMOF	MgMOF-Cur	MgMOF-Cur at 40 °C
% crystallinity	84.8	65.6	81.0	84.2

The MgMOF-Cur prepared at room temperature exhibited a crystallinity of 81.0%, while the sample prepared by heating to 40 °C exhibited an increased crystallinity of 84.2%, approaching that of free curcumin (84.8%), supporting our previous suggestion of the surface adsorption of curcumin on the external surface of the framework, allowing it to significantly contribute to the overall crystallinity.

The third parameter investigated was the MOF:Curcumin molar ratio. This was done by preparing three samples with different molar ratios of 1:2, 1:4, and 1:6. Among them, the 1:4 ratio yielded the highest loading efficiency (46 wt %) and was therefore selected as the optimal loading ratio.

### 3.2 Characterization of the MgMOF-Cur System by SEM and FTIR

Based on the SEM images (Figure 4), as compared to the spherical shape of the bare MgMOF (Figure 4B), bigger aggregations were observed for MgMOF-Cur (Figure 4C), confirming the successful immobilization of curcumin and suggesting its potential bridging role, where it is promoting the association of the MOF particles by coordinating to multiple particles and acting as a bridging ligand.

**Figure 4:**
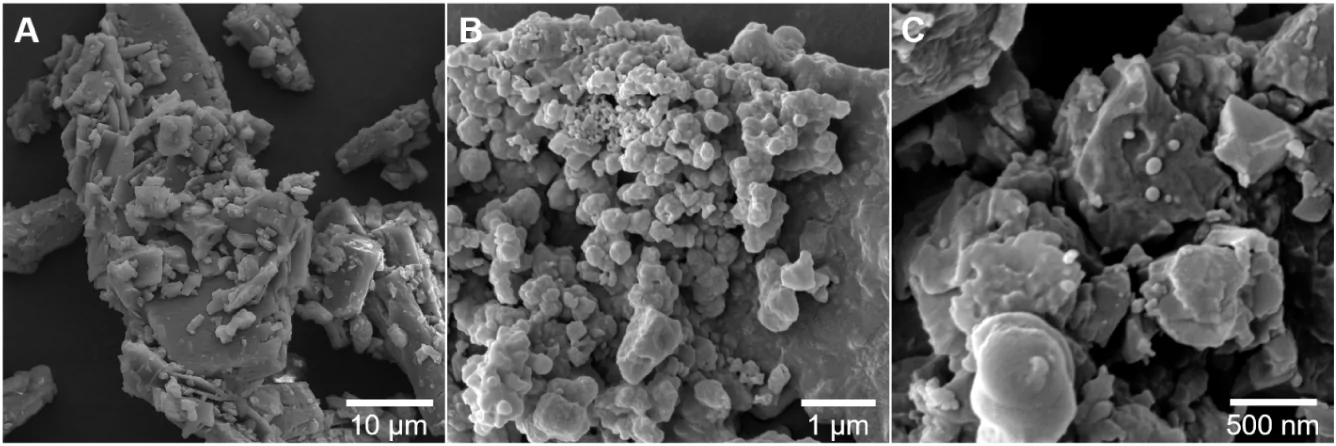
SEM images of (A) pure curcumin; (B) magnesium formate; and (C) curcumin-loaded magnesium formate MOF stirred for 1 h at 40 °C.

These observations are further supported by FTIR analysis, which was performed to investigate the main interactions between the functional groups of curcumin and the surface of the MgMOF.

As shown in Figure 5, the spectrum of MgMOF displayed four characteristic bands at 2910, 1685, 1357, and 767 cm^–1^. These wavenumbers are attributed respectively to the C–H stretching, asymmetric and symmetric O–C–O vibrations, and O–C–O symmetric bending.^38^ The FTIR spectrum of the MgMOF-Cur composite retained the characteristic bands of curcumin: 3508 cm^–1^ (phenolic OH), 1631 cm^–1^ (C–C and C=O), 1600 cm^–1^ (C–C_ring_ stretching), 1518 cm^–1^ (C=O), 1261 cm^–1^ (aromatic C–O), and 1019 cm^–1^ (C–O–C),^39^ along those of the MgMOF, confirming the successful immobilization of curcumin on the surface of the MOF. Interestingly, both the peaks assigned to the phenolic OH and the aromatic CO vibrations of curcumin were broadened when loaded onto MgMOF suggesting an interaction between these phenolate groups of curcumin and the Mg centers in the MOF. This interaction may involve partial substitution of the surface formate ligands in MOF by the phenolate groups of curcumin, allowing it to act as a bridging molecule between adjacent Mg centers, which is consistent with the SEM results showing particle aggregation.

**Figure 5:**
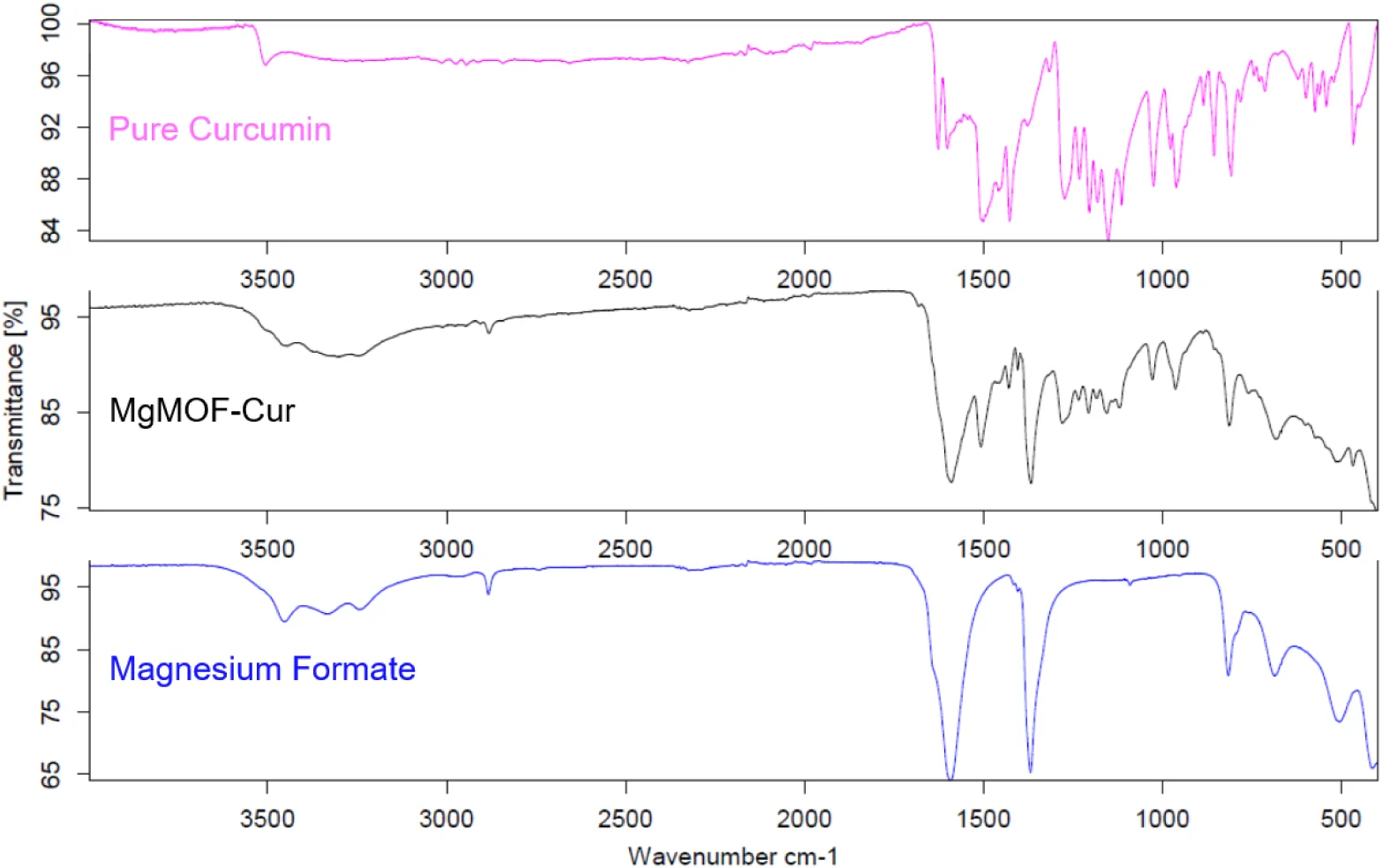
FTIR spectra of pure curcumin, magnesium formate, and MgMOF-Cur.

### 3.3 Effect of Curcumin Adsorption on Its Stability over Time

The main purpose of curcumin loading onto MgMOF was to enhance its chemical stability, as curcumin is known to readily degrade in aqueous solution. To evaluate this effect, the absorption spectrum of an MgMOF-Cur solution was monitored over 10 days and compared with that of pure curcumin.

As shown in Figure 6, a substantial decrease in the absorbance of free curcumin was observed after only 3 days, corresponding to ∼58.5% degradation. In contrast, curcumin adsorbed onto the surface of MgMOF exhibited a 4-fold lower degradation (∼16.0%), significantly maintaining its stability over time. These findings align with previous reports demonstrating enhanced aqueous stability of curcumin upon incorporation into magnesium-based MOFs.^40^ After 10 days the % degradation of the MgMOF-Cur system increased to 33.7% but remained lower than that of pure curcumin after 3 days. These results demonstrate the successful stabilization of curcumin through loading into the biocompatible Mg-formate MOF.

**Figure 6:**
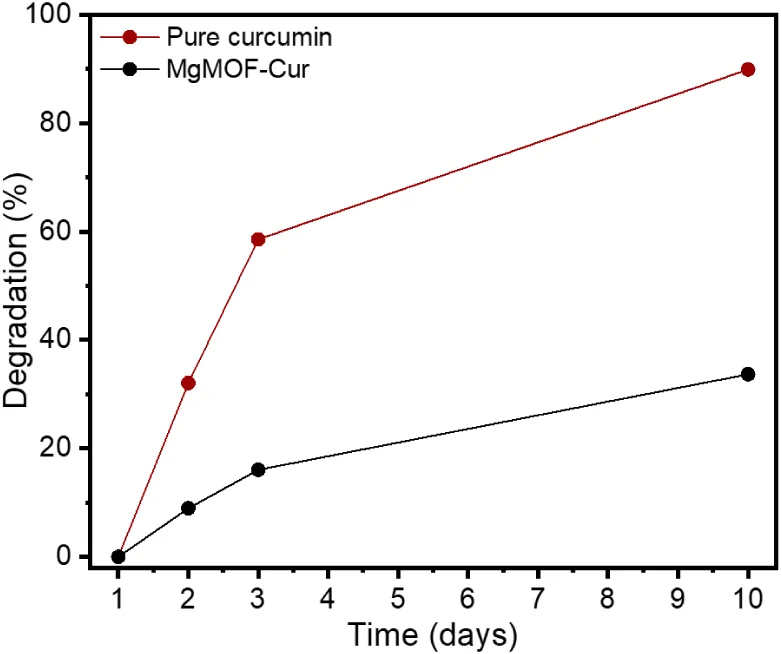
Time-dependent degradation of free curcumin and MgMOF-Cur.

### 3.4 Scavenging Effect of Curcumin

The effect of curcumin immobilization onto MgMOF on its antioxidant activity was evaluated using the DPPH radical scavenging assay. This method consists of using the DPPH radical which has an unpaired electron on its nitrogen, giving it a high tendency to abstract a hydrogen from the phenolic OH of curcumin forming a phenoxy radical.^41^

Upon adding a fixed concentration of DPPH (0.04 mg/mL) into increasing concentrations of MgMOF-Cur solutions (0.01 mg/mL to 0.04 mg/mL), the absorbance was measured at 540 nm and the scavenging activity was calculated according to the equation below:

$$\%Scavenging=\frac{A_{DPPH}-A_{S}}{A_{DPPH}}\times 100$$

where A_DPPH_ refers to the initial absorbance of DPPH and A_S_ represents the absorbance after reaction with MgMOF-Cur for an hour.

As shown in Figure 7A, DPPH absorbance decreased with increasing concentration of MgMOF-Cur. This can be better visualized in Figure 7B, where the absorbance of DPPH at 540 nm decreased by ∼65% upon the addition of 0.04 mmol of MgMOF-Cur. This decrease in absorbance is attributed to the reduction of the DPPH radical (purple) into DPPH-H (yellow) due to the H-abstraction from curcumin (Figure 7C).

**Figure 7:**
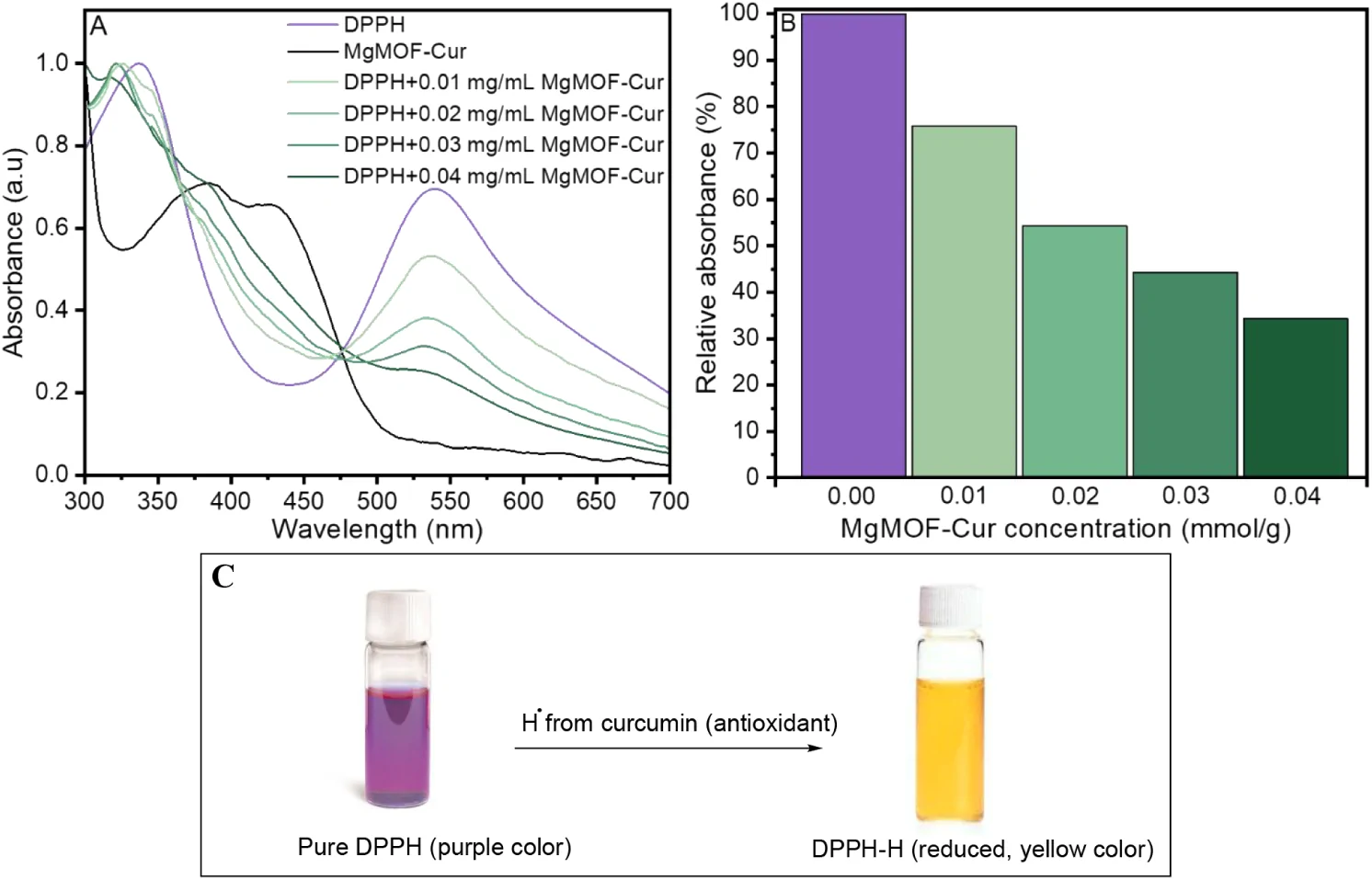
(A) Effect of increasing MgMOF-Cur concentrations (0.01 mg/mL to 0.04 mg/mL) on the absorbance of DPPH radical; (B) percentage decrease in the absorbance of DPPH at 540 nm with increasing concentrations of MgMOF-Cur; and (C) representative scheme illustrating the color change of the DPPH solution before and after the addition of MgMOF-Cur (the scheme was drawn by the author using canva draw based on the color obtained experimentally).

Based on the data, the scavenging percentage was calculated and shown in Table 2, revealing a direct correlation between the concentration of MgMOF-Cur and radical scavenging efficiency, confirming the preservation of curcumin’s antioxidant properties, which aligns with earlier findings in similar systems.^42^

**Table 2:** Scavenging Activity of the MgMOF-Cur System in the Concentration Range of 0.01 mg/mL to 0.04 mg/mL under Neutral pH

Concentration (mg/mL)	0.01	0.02	0.03	0.04
Scavenging %	16	35	40	47

Moreover, since curcumin exists in different structural forms depending on the pH of the medium,^43^ the scavenging activity of the MgMOF-Cur system was assessed at pH = 4 and pH = 10. No scavenging activity was observed in acidic medium as curcumin exists in its diketo form, which limits its interaction with the DPPH radical.^44^ Therefore, the test was limited to pH = 7 and pH = 10, and the results are presented in Table 3.

**Table 3:** Effect of pH (7 and 10) on the Scavenging Activity of the MgMOF-Cur System

Sample concentration (mg/mL)	Scavenging activity (%) at pH = 7 (*n* = 3)	Scavenging activity (%) at pH = 10 (*n* = 3)
0.01	16	12
0.02	35	17
0.03	40	28
0.04	47	33

As shown, curcumin exhibited higher scavenging in neutral medium due to the presence of the enol tautomer of curcumin, allowing the highly reactive OH to induce the reduction of the DPPH radical. On the other hand, the lower scavenging activity observed under basic conditions can be attributed to the deprotonation of the phenolic hydroxyl groups, limiting the H donation to the DPPH radical. The mechanism of DPPH scavenging with curcumin in its enol and keto forms is illustrated in Scheme 2.

**Scheme 2:**
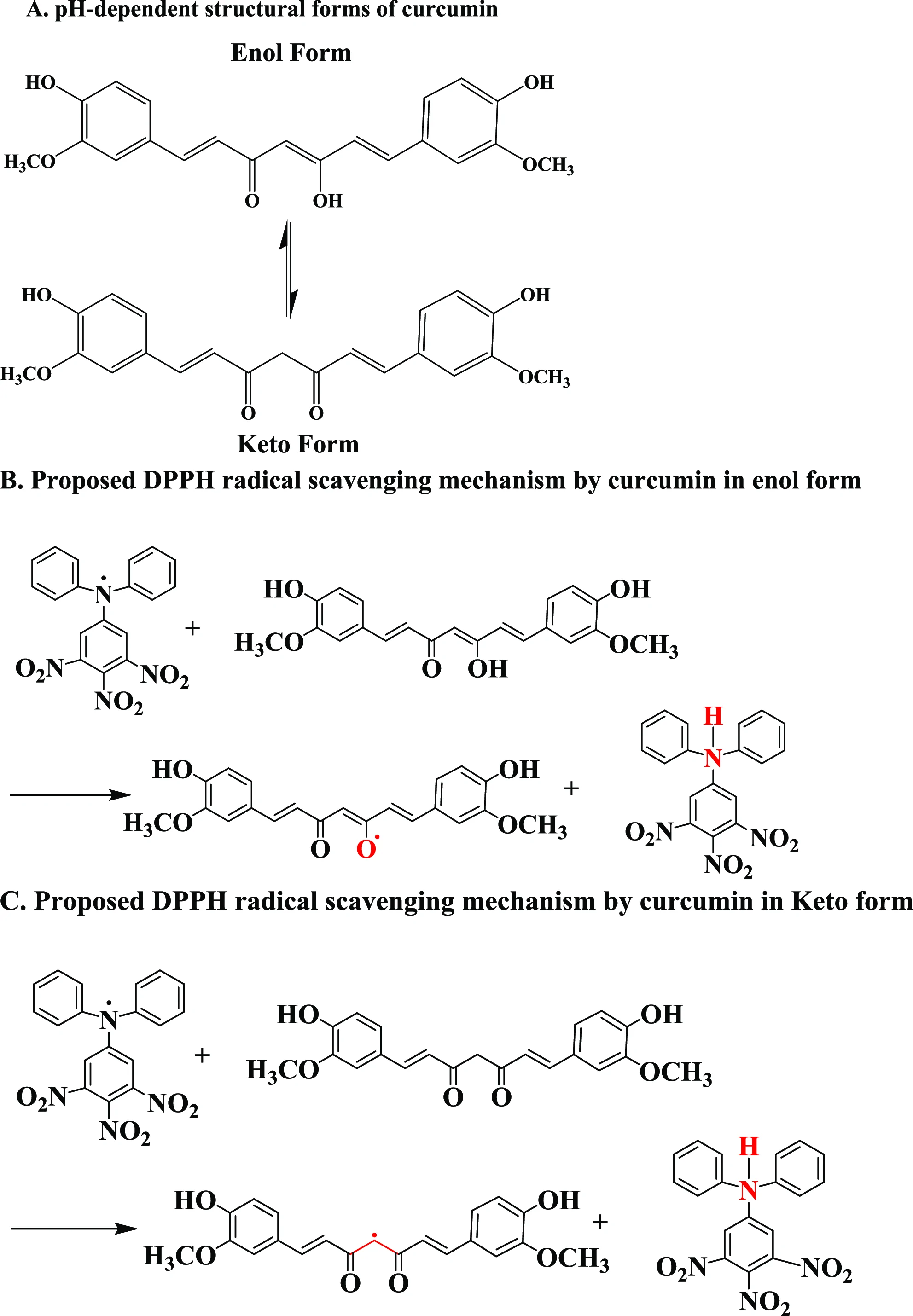
Tautomerization of Curcumin and Its Mechanism with DPPH in Scavenging Activity

Furthermore, to evaluate the effect of curcumin loading onto MgMOF, the radical scavenging activity of the MgMOF-Cur system was compared with that of free curcumin. The results shown in Table 4 reveal a substantially higher scavenging activity than free curcumin at all investigated concentrations, with the scavenging percentage reaching 47% compared with only 13% for free curcumin at 0.04 mg/mL.

**Table 4:** Comparison of the Scavenging Activity of the MgMOF-Cur System and Free Curcumin, in the Concentration Range of 0.01 mg/mL to 0.04 mg/mL under Neutral pH

Sample concentration (mg/mL)	Scavenging activity of MgMOF-Cur (%) (*n* = 3)	Scavenging activity of free curcumin (%) (*n* = 3)
0.01	16	0.5
0.02	35	4
0.03	40	11
0.04	47	13

## 4 Conclusion

In this study, curcumin was successfully immobilized onto the surface of MgMOF using a simple wet impregnation method, and the loading parameters were systematically optimized. A stirring time of 1 h at 40 °C and a MgMOF:curcumin molar ratio of 1:4 were identified as optimal conditions, leading to enhanced thermal stability and higher loading efficiency without compromising the structural integrity of the framework. Characterization by TGA, PXRD, SEM, and FTIR confirmed the successful loading of curcumin and suggested interactions between curcumin and the Mg centers of the MOF. Importantly, the MgMOF-Cur system significantly improved curcumin’s stability in aqueous environments, reducing its degradation over time compared with free curcumin. Furthermore, the antioxidant activity of curcumin was preserved upon loading onto MgMOF, as demonstrated by DPPH radical scavenging assays under different pH conditions. These findings demonstrate that MgMOF is a promising carrier for improving the aqueous stability and antioxidant performance of curcumin. However, further biological investigations, including cytotoxicity, cellular uptake, and therapeutic efficacy studies, are necessary to validate its potential for biomedical and therapeutic applications.

## #

Pamela Al Azzi: Normandie University, ENSICAEN, UNICAEN, Catalysis and Spectrochemistry Laboratory (LCS), 14050 Caen, France

## ∇

Joelle Mesmar: American University of Beirut, Department of Dentofacial Medicine, P.O. Box 11-0236, Riad El-Solh, Beirut 1107 2020, Lebanon.
